# Local Delivery of Taxol From FGL-Functionalized Self-Assembling Peptide Nanofiber Scaffold Promotes Recovery After Spinal Cord Injury

**DOI:** 10.3389/fcell.2020.00820

**Published:** 2020-08-21

**Authors:** Zhiyong Xiao, Yingtao Yao, Zhiyu Wang, Qing Tian, Jiedong Wang, Li Gu, Bo Li, Qixin Zheng, Yongchao Wu

**Affiliations:** ^1^Department of Orthopaedics, Union Hospital, Tongji Medical College, Huazhong University of Science and Technology, Wuhan, China; ^2^Wuhan National Laboratory for Optoelectronics, Britton Chance Center for Biomedical Photonics, Huazhong University of Science and Technology, Wuhan, China; ^3^MoE Key Laboratory for Biomedical Photonics, Collaborative Innovation Center for Biomedical Engineering, School of Engineering Sciences, Huazhong University of Science and Technology, Wuhan, China; ^4^Department of Pathology, Zhongnan Hospital of Wuhan University, Wuhan, China; ^5^Department of Orthopaedics, The First Affiliated Hospital of Zhengzhou University, Zhengzhou, China; ^6^Department of Orthopaedics, Beijing Jishuitan Hospital, Beijing, China

**Keywords:** Taxol, spinal cord injury, neurite regeneration, self-assembling peptides, FGL, drug release, microtubule stabilization

## Abstract

Taxol has been clinically approved as an antitumor drug, and it exerts its antitumor effect through the excessive stabilization of microtubules in cancer cells. Recently, moderate microtubule stabilization by Taxol has been shown to efficiently promote neurite regeneration and functional recovery after spinal cord injury (SCI). However, the potential for the clinical translation of Taxol in treating SCI is limited by its side effects and low ability to cross the blood-spinal cord barrier (BSCB). Self-assembled peptide hydrogels have shown potential as drug carriers for the local delivery of therapeutic agents. We therefore hypothesized that the localized delivery of Taxol by a self-assembled peptide scaffold would promote axonal regeneration by stabilizing microtubules during the treatment of SCI. In the present study, the mechanistic functions of the Taxol-releasing system were clarified *in vitro* and *in vivo* using immunofluorescence labeling, histology and neurobehavioral analyses. Based on the findings from the *in vitro* study, Taxol released from a biological functionalized SAP nanofiber scaffold (FGLmx/Taxol) remained active and promoted neurite extension. In this study, we used a weight-drop contusion model to induce SCI at T9. The local delivery of Taxol from FGLmx/Taxol significantly decreased glial scarring and increased the number of nerve fibers compared with the use of FGLmx and 5% glucose. Furthermore, animals administered FGLmx/Taxol exhibited neurite preservation, smaller cavity dimensions, and decreased inflammation and demyelination. Thus, the local delivery of Taxol from FGLmx/Taxol was effective at promoting recovery after SCI and has potential as a new therapeutic strategy for SCI.

## Introduction

Spinal cord injury (SCI) is typically followed by functional deficiencies below the level of the lesion site. Compared with the peripheral nervous system, neurons in the adult central nervous system (CNS) do not normally display axonal regeneration after injury (Harel and Strittmatter, 2006; Mahar and Cavalli, 2018). Many mechanisms contribute to the failure of axonal regeneration, including reactive gliosis and inflammation, insufficient intrinsic growth potential of mature neurons and the presence of inhibitory factors, such as chondroitin sulfate proteoglycans (CSPGs), at the lesion site (Filbin, 2003; Yiu and He, 2006; Liu et al., 2011). Although many therapeutic approaches have been applied to improve the regenerative capability of injured axons (Sun et al., 2018; Courtine and Sofroniew, 2019; Koffler et al., 2019), these approaches are still far from successful translation into clinical practice. Therefore, strategies that easily translate from experimental treatments to clinical treatments for SCI have been highlighted by many neurobiologists in the nerve regeneration field.

Taxol, an FDA-approved anticancer drug, alters microtubule acetylation and promotes microtubule stabilization (Prota et al., 2013). Taxol remarkably increases axonal growth after SCI and protects cultured neurons from axonal retraction following axotomy (Baas et al., 2016; Fan et al., 2018; Yin et al., 2018). Moreover, Taxol also restores the axonal growth potential in the presence of inhibitory molecules (e.g., Nogo-A and CSPGs) (Sengottuvel et al., 2011). In addition, Taxol inhibits the formation of fibrotic scars at the lesion site by blocking TGF-β signaling (Hellal et al., 2011). Although microtubule stabilization by Taxol is very promising for clinical applications to treat CNS injury, the delivery system is limited by the properties of Taxol, such as hydrophobicity, non-specific binding, narrow treatment window, and large molecular size, which prevents it from crossing the blood-spinal cord barrier (BSCB) (Brunden et al., 2011). The effective concentration range of Taxol for promoting nerve regeneration is extremely narrow. At higher concentrations, Taxol-mediated neurite elongation in mature primary neurons was reduced, even in the presence of microtubule hyperstabilization (Sengottuvel et al., 2011). One tested delivery approach involved directly applying Taxol to the injured site of the spinal cord using an osmotic minipump (Popovich et al., 2014). This approach is not feasible for treating patients, as these surgically implanted minipumps can be dislodged, can induce infections and have a limited loading capacity. Therefore, the design of a feasible localized delivery system for the clinical application of Taxol in treating SCI is important. Biomaterial-based drug carriers, such as self-assembled peptide (SAP) hydrogels, show promise in the delivery of Taxol to the lesion site and promotion of spinal cord tissue repair.

In recent decades, hydrogels have been developed as delivery systems for proregenerative agents to promote neural tissue regeneration after SCI (Hassannejad et al., 2019; Ma et al., 2020; Nazemi et al., 2020). According to several studies, synthetic SAP hydrogels have been designed and form nanofiber scaffolds *in vivo* under physiological conditions (Ahuja and Fehlings, 2016; Koss and Unsworth, 2016; Hong et al., 2017). These properties make SAP hydrogels non-toxic and degradable, with excellent biocompatibility and low apparent immunogenicity, and thus they are feasible for application in the treatment of SCI via direct injection of the peptide solution into the lesion site. In our previous study (Wang et al., 2015), We synthesized a new RADA_16_-FGL peptide (AcNRADARADA RADARADAGGEVYVVAENQQGKSKA-CONH_2_) containing FGL (EVYVVAENQQGKSKA), the motif of neural cell adhesion molecule (NCAM), designed a new biological functionalized SAP nanofiber scaffold named FGLmx by assembling RADA-FGL with the pure RADA_16_ peptide (AcN-RADARADARADARADA-CONH_2_), assessed its physiochemical characteristics, and evaluated its biocompatibility with and effects on neural stem cells (NSCs). FGLmx self-assembles into nanofiber scaffolds and promotes NSC adhesion and survival. Furthermore, FGLmx provides better support for NSC proliferation and migration into the scaffold than RADA_16_ alone. Based on these results, biologically functionalized SAP hydrogels might be a beneficial choice for neural tissue engineering applications.

In the current study, we loaded Taxol into FGLmx and tested the hypothesis that the administration of the Taxol delivery system, i.e., FGLmx/Taxol, promotes neurite elongation *in vitro* and *in vivo*. We subsequently characterized the capability of neurites to overcome the challenges that occur in traumatic SCI when treated with the Taxol-loaded scaffold. The injection of the FGLmx/Taxol delivery system immediately after SCI might be effective at promoting recovery and neural regeneration after SCI.

## Materials and Methods

### Fabrication of Taxol-Loaded Nanofiber Scaffold

RADA_16_ and RADA_16_-FGL were synthesized with F-mocchemistry using automated solid-phase peptide synthesis at a biopharmaceutical firm (Chinese Peptide Company, Hangzhou) and purified by high-performance liquid chromatography (HPLC). Pure RADA_16_ and RADA_16_-FGL powders were mixed at a weight ratio of 1:1. The mixed peptide powders were dispersed in sterile distilled water at a concentration of 1% (w/v) to obtain the novel peptide scaffold named FGLmx and subsequently sonicated for 30 min prior to use. The Taxol (Sigma, United States) powder was mixed with FGLmx solution at a desired concentration of 1% (w/v). Then, the Taxol-loaded peptide solution was stirred and incubated overnight at room temperature, resulting in the formation of the Taxol-loaded FGLmx hydrogel, which was subsequently used in later drug release experiments.

### Characterization of FGLmx/Taxol Nanofiber Scaffold

We used atomic force microscopy (AFM) to study the morphology of the self-assembling scaffold, as described in a previous study (Genove et al., 2005). Briefly, 10 μL of the 1% (w/v) peptide solution (FGLmx or FGLmx/Taxol) were deposited on a clean mica surface, incubated for 30 s, rinsed with distilled water three times and then air-dried. AFM images were captured with a scanning probe microscope (Nanoscope IIIa, Digital Instruments, CA) in tapping mode. The root mean square (RMS) amplitude was determined before engaging at a voltage of 1–1.2 V, a tapping frequency of 75 kHz, set point of 0.7–1.0 V and scanning speed of 1.4 Hz.

### Drug Release Profile *in vitro*

One hundred microliters of FGLmx/Taxol hydrogel were incubated with 900 μL of phosphate-buffered saline (PBS, Gibco, United States) containing 0.1% (w/v) Tween 80 and 0.02% (w/v) NaN_3_ to determine the profile of Taxol release from the FGLmx hydrogel. The Taxol-loaded hydrogel was then placed in a rotary shaker and incubated at 50 rpm and 37°C. An aliquot of 500 μL of supernatant containing the released Taxol was removed, stored at −20°C, and replaced with the same amount of fresh PBS at the following desired time points: 1, 3, 6, 9, 12, 15, 18, 21, 24, 27, and 30 days. The Taxol concentration in the supernatant was measured using HPLC and is presented as the cumulative amount of Taxol released over time.

### Bioactivity Assessment of Released Taxol *in vitro*

The supernatants of FGLmx and FGLmx/Taxol were collected at d 3 to assess the bioactivity of Taxol released from the hydrogel. All animal experiments were performed in accordance with the Guide for the Care and Use of Laboratory Animals (Clark et al., 1997). All animal procedures were also reviewed and approved by the Hubei Provincial Animal Care and Use Committee and complied with the experimental guidelines of the Animal Experimentation Ethics Committee of Huazhong University of Science and Technology in China. Primary rat cortical neurons were isolated from neonatal (P0) Sprague-Dawley (SD) rats (obtained from the Animal Center of Tongji Medical College, Huazhong University of Science and Technology) using a previously established procedure (Zille et al., 2017). The primary neurons were cultured in 6-well plates in Neurobasal Medium (Gibco, United States) supplemented with 1% (v/v) fetal bovine serum (FBS, Gibco, United States), 2% (v/v) B-27 serum-free supplement (Gibco, United States), 1% penicillin–streptomycin (Beyotime, China) and 1% L-glutamine (Sigma, United States) at 37°C in a 5% CO_2_ atmosphere. For the experimental group, the cells were cultured with medium containing the Taxol-loaded supernatant on the next day. According to the HPLC analysis, the concentration of Taxol in the medium was 4 nM. For the other experimental group, the cells were cultured with medium containing supernatant of FGLmx. For consistency, We maintained the same concentration of FGLmx in the medium of both experimental groups. The control group was incubated with 0.5 mL of PBS without Taxol. The medium was changed every 2 d.

### *In vitro* Immunocytochemistry

After culture *in vitro* for 72 h, the primary neurons were fixed with a 4% paraformaldehyde solution (Guge Biotech, Wuhan, China) for 15 min. Then, the cells were prepared for immunostaining with a mouse anti-βIII tubulin antibody (TUJ-1, 1:1000, Abcam, United States) at 4°C overnight and subsequently stained with an Alexa Fluor 488-conjugated goat anti-mouse secondary antibody (1:200, Santa Cruz, CA) at room temperature for 1.5 h. All neurites in wells were visualized using a fluorescence microscope (Olympus BX53, Japan). Neurite extension from the cortical neurons was quantified using ImageJ software (NIH, United States). The mean neurite length was obtained as the sum of the lengths of neurites per well averaged by the total number of βIII tubulin-positive neurites per well.

### Spinal Cord Contusion and Intraspinal Microinjection of the Hydrogel Scaffold in Rats

**FIGURE 1:**
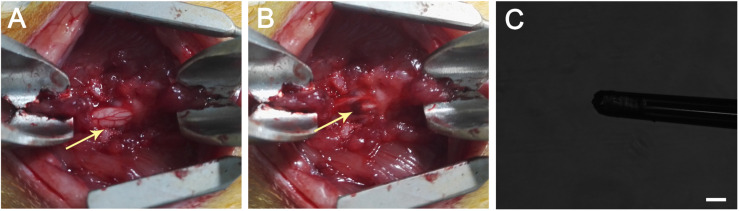
**(A)** T9 laminectomy segment. **(B)** A hematoma occurred after the dorsoventral contusion of the spinal cord using a weight-drop impactor, which generated a 250-kDyne contusion injury. The arrow head indicates the hematoma after SCI. **(C)** Injection performed using a glass capillary needle (the diameter is less than 100 μm) connected to a micromanipulator of the stereotactic apparatus. The scale bar represents 100 μm.

In this experiment, the thoracic SCI model was established as previously reported (Nicola et al., 2019). Briefly, 30 adult female SD rats weighing 200–220 g were obtained from the Animal Center of Tong Ji Medical College, Huazhong University of Science and Technology. We randomly divided the 30 animals into the following three groups: 1. animals treated with the FGLmx hydrogel (FGLmx group, *n* = 10); 2. animals treated with the Taxol-loaded FGLmx hydrogel (FGLmx/Taxol group, *n* = 10); and 3. animals treated with 5% glucose (control group, *n* = 10). All rats were anesthetized with an intraperitoneal injection of 10% chloral hydrate (400 mg/kg, Sigma, United States). After a T9 laminectomy, an NYU-MASCIS weight-drop impactor with a 10-g rod dropped from a height of 25 mm was used to create a 250-kDyne contusion injury at the T9 level of the spinal cord. After the contusion injury, each animal was placed in a stereotaxic frame, and 10 μL of the 1% (w/v) peptide solution containing FGLmx alone or FGLmx/Taxol were injected into the epicenter of the injury site using a glass capillary needle fixed to a micromanipulator (Figure 1). An identical amount of 5% glucose was injected into animals in the control group using the same procedure. Subsequently, the muscles were sutured in layers, and the skin was closed. Animals received the antibiotic amoxicillin (10 mg/kg, Sinopharm, China) daily for 1 week after surgery to prevent infection. Food and water were available *ad libitum*. The bladder was manually voided twice a day until the voiding reflex was restored.

### Neurobehavioral Tests

The neurobehavioral recovery of the rats after SCI was evaluated using the Basso, Beattie, and Bresnahan (BBB) score (Basso et al., 1996; Lebedev et al., 2008). Voluntary movements of the animals were evaluated at specific time points by two independent experimenters who were blinded to the protocol. The scale (Supplementary Table S3) included hindlimb joint movements, stepping ability, forelimb-hindlimb coordination and trunk stability. Animals were placed in the center of an open floor, and the scores were recorded strictly according to the observations. Each animal was observed and the score was recorded every week for no less than 4 min. Scores for each animal were obtained by averaging the values of the right and left hindlimbs.

### *In vivo* Histology and Immunofluorescence Staining

Eight weeks after injury, animals were anesthetized with an overdose of 10% chloral hydrate and subsequently sacrificed. Each animal was transcardially perfused with ice-cold 0.9% saline (pH 7.3) for 5 min followed by 4% paraformaldehyde (Guge Biotech, Wuhan, China) in PBS (pH 7.3) for 6 min. The thoracic spinal cord segments were carefully collected, post fixed with 4% paraformaldehyde at 4°C overnight, dehydrated in 30% sucrose and embedded in optimal cutting temperature (OCT) compound prior to cryosectioning. A series of longitudinal sections (10-μm thick) were collected and stored at −20°C prior to hematoxylin and eosin (H&E) staining for a histological assessment of the lesion volume and tissue sparing.

Immunofluorescence staining was conducted using standard protocols, as previously described (Zweckberger et al., 2016). Slices were incubated with primary antibodies, including anti-glial fibrillary acidic protein (GFAP; 1:400; Abcam; United States) for astrocytes, anti-neurofilament 200 (NF200; 1:1000; Abcam; United States) for axons, anti-myelin basic protein (MBP; 1:1000; Abcam; United States) for myelin sheath, and anti-CD68 (1:100; Santa Cruz; United States) for macrophages, overnight at 4°C. After three washes with PBS, the slices were incubated with secondary antibodies conjugated with Alexa Fluor 546 (1:300; Abcam; United States) or Alexa Fluor 488 (1:300; Abcam; United States). After a 2-h incubation at room temperature, all specimens were washed with PBS and subsequently counterstained with 4′,6-diamidino-2-phenylindole (DAPI; Guge Biotech, Wuhan, China). All brightfield and fluorescence images of the histological slices were visualized and captured using a confocal microscope (Zeiss, Inc., Germany), followed by quantification using ImageJ software.

### Luxol Fast Blue (LFB) Staining

After the final BBB score was obtained, the injured spinal cord was harvested and prepared as previously described (Yang and Jou, 2016; Geissler et al., 2018). Serial transverse sections with a thickness of 5 μm were prepared from the embedded blocks, and slices were collected at a 3 mm interval. Five slices in the rostral to caudal direction were collected from each sample and stained using previously described methods (Yang and Jou, 2016). Briefly, sections were deparaffinized with xylene (Sigma, United States) and rehydrated in a series of ethanol solutions (100, 95, and 70%) followed by distilled water (Millipore, United States). Then, the spinal cord sections were stained with the LFB solution (Sigma, United States) overnight at 60°C to analyze the extent of myelin sparing at the lesion epicenter. Excess dye was removed with 95% ethanol, and the sections were subsequently washed with distilled water three times. The sections were then destained with a lithium carbonate solution (0.05%, Sigma, United States) for 30 s and washed with distilled water, followed by dehydration in 95% ethanol once and 100% ethyl alcohol (Sigma, United States) twice. Finally, the sections were mounted with neutral resin and dried at room temperature for 5 min. Five sections from the spinal cord of each animal, including sections from the lesion epicenter, were assessed for LFB-positive areas. Bright field images were obtained with an Olympus BX53 microscope. By determining the integrated optical density (IOD) of the spared myelin sheath in the selected area, the spared myelin area in the posterior horn of the spinal cord was quantified using software (Image-Pro Plus 6.0).

### Statistical Analysis

All data are presented as means ± SD, and SPSS 20.0 software for Windows was used to perform the analyses. Significant differences between groups were determined using one-way ANOVA, followed by the Student-Newman-Keuls *post hoc* test. Differences were considered statistically significant at *P* < 0.05.

## Results

### SAP Synthesis and Scaffold Fabrication

SAPs were prepared as reported in our previous study (Wang et al., 2015). RADA_16_ and the FGL-functionalized peptide (RADA_16_-FGL) were successfully analyzed using HPLC and mass spectrometry (MS). The molecular weights of RADA_16_ and RADA_16_-FGL were 1712 with a purity of 99.30% and 3458 with a purity of 99.46%, respectively.

### Hydrogel Formation and Design and Characterization of the FGLmx/Taxol Self-Assembling Nanofiber Scaffold for Controlled Drug Release

**FIGURE 2:**
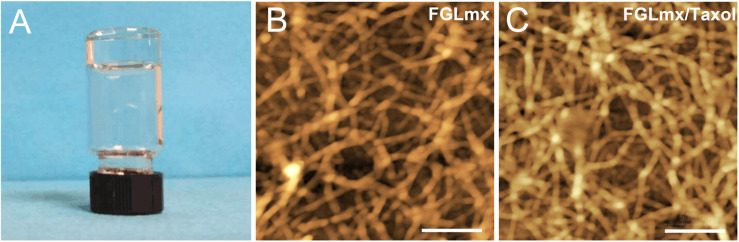
**(A)** The FGLmx/Taxol peptide solution formed a transparent gel in PBS; the peptide concentration was 1% (w/v). AFM images of the ultramicrostructures of the FGLmx **(B)** and FGLmx/Taxol **(C)** nanofiber scaffolds. The scale bar represents 500 nm.

The FGLmx/Taxol powder was dissolved in ddH_2_O at a concentration of 1% (w/v), stirred overnight at room temperature, and subsequently dissolved in PBS to prepare the hybrid hydrogel controlled release system, which allowed the peptides to self-assemble and form a stable transparent gel (Figure 2A). An examination of the ultramicrostructure of the gel using AFM showed that nanofibers formed after FGLmx self-assembly. High densities of interwoven nanofiber structures were observed in both the FGLmx and FGLmx/Taxol groups (Figures 2B,C). The diameters of the fibers assembled from FGLmx and FGLmx/Taxol were 16.5 ± 1.2 and 25.3 ± 1.8 nm, respectively. The difference in the length of fibers between the FGLmx and FGLmx/Taxol groups was not statistically significant, indicating that loading Taxol into FGLmx did not alter the arrangement of the nanofiber structures.

### Taxol Release *in vitro*

**FIGURE 3:**
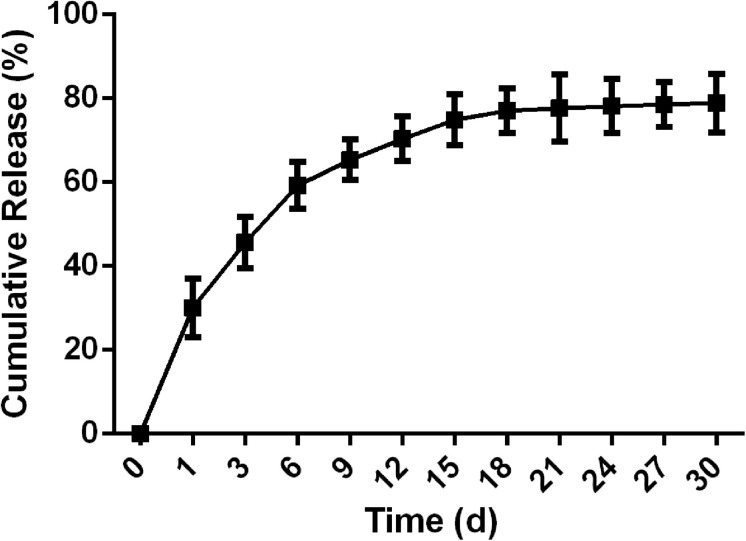
Cumulative release of Taxol from FGLmx/Taxol *in vitro*.

The release of Taxol from the FGLmx peptide hydrogel under physiological conditions was analyzed using HPLC. As shown in Figure 3, the *in vitro* release characteristics included a burst release of Taxol in the first week, followed by sustained release over the next 30 d. Thus, 75% of the Taxol incorporated into the hydrogel was released from the nanofiber scaffold.

### Released Taxol Remained Active and Promoted Neurite Extension *in vitro*

**FIGURE 4:**
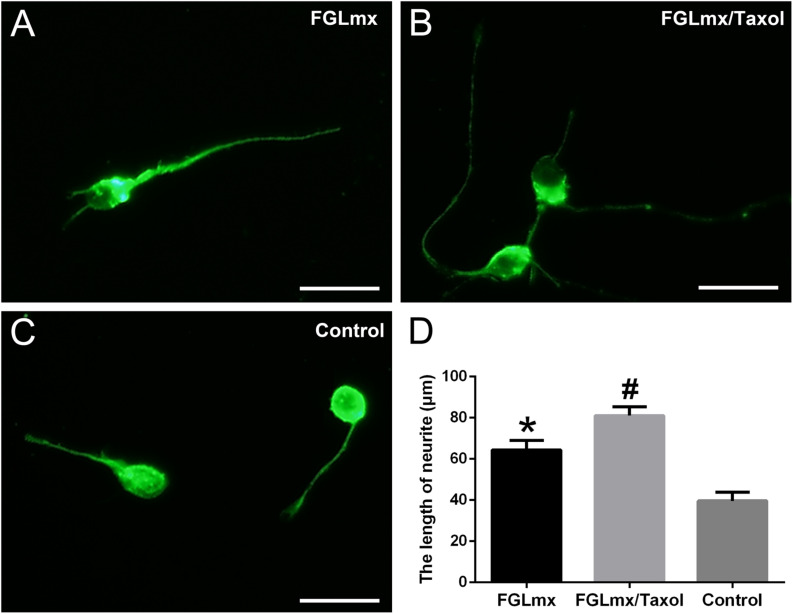
The administration of Taxol released from FGLmx/Taxol increased neurite outgrowth from primary cortical neurons *in vitro*. βIII-Tubulin (green) staining of primary cortical neurons after 3 days of culture with **(A)** the supernatant of FGLmx, **(B)** supernatant of FGLmx/Taxol, or **(C)** PBS as a control. The scale bar represents 25 μm. **(D)** Quantification of the neurite length per well (± SEM). ^#^*P* < 0.05 compared with the other two groups. *P < 0.05 compared with the control group.

We cultured primary rat cortical neurons with the supernatant of FGLmx/Taxol and FGLmx *in vitro* to determine whether the released Taxol retained its bioactivity. The addition of Taxol-loaded supernatant significantly promoted neurite outgrowth (81.00 ± 1.924 μm, Figure 4B) compared with the supernatant of the FGLmx-treated group (64.40 ± 2.040 μm, Figure 4A). However, primary neurons in the control group showed moderate neurite outgrowth (39.60 ± 1.860 μm, Figure 4C), suggesting that Taxol remained active and promoted neurite outgrowth after release from the FGLmx/Taxol drug delivery system.

### The FGLmx/Taxol Treatment Increased Neurofilament Preservation

**FIGURE 5:**
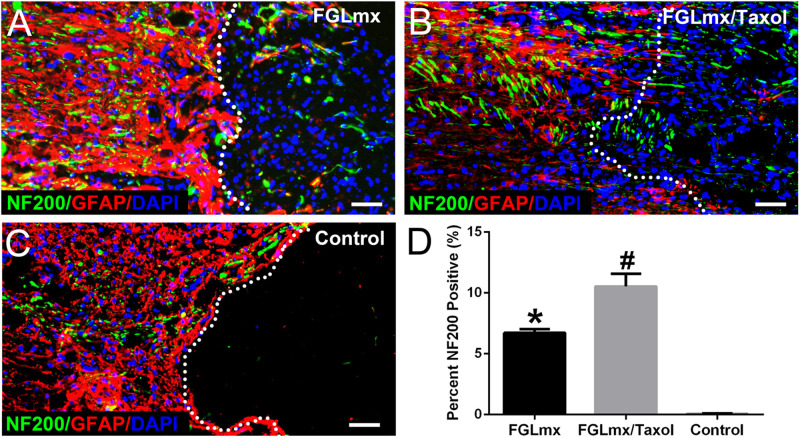
Presence of neurites (green) extending beyond the lesion border in longitudinal sections containing the lesion epicenter: GFAP (red), NF200 (green), and DAPI (blue). Animals were treated with FGLmx **(A)**, FGLmx/Taxol **(B)**, and 5% glucose as the control **(C)**. The white dotted line indicates the lesion border. The scale bar represents 50 μm. **(D)** Quantification of neurites extending beyond the lesion border (± SEM). ^#^*P* < 0.05 compared with the other two groups; *P < 0.05 compared with the control group.

The presence of NF200-positive (neurofilament 200, a marker of neurons and axons) neurofilaments across the injury site was quantified to evaluate the effect of FGLmx/Taxol on neurite regeneration *in vivo*. The administration of the SAP scaffold increased the density of NF200-positive neurofilaments at the injury site compared with the administration of 5% glucose. Moreover, animals treated with FGLmx/Taxol exhibited an increase in the number of neurofilaments at the injury site compared with animals treated with FGLmx (*P* < 0.05). At 8 weeks after injury, animals in the FGLmx/Taxol-treated group showed an average neurofilament density of 10.53 ± 0.460% within the injury site (Figure 5B). However, animals in the FGLmx-treated group and the 5% glucose-treated control group showed a neurofilament density of 6.710 ± 0.140% (Figure 5A) and 0.050 ± 0.017% (Figure 5C), respectively. The addition of Taxol appeared to increase the preservation of neurofilaments. Based on these data, the local delivery of Taxol from the FGL-functionalized SAP scaffold increased neurite regeneration at the injury site following SCI.

### The FGLmx/Taxol Treatment Reduced Astrogliosis and Inflammation

**FIGURE 6:**
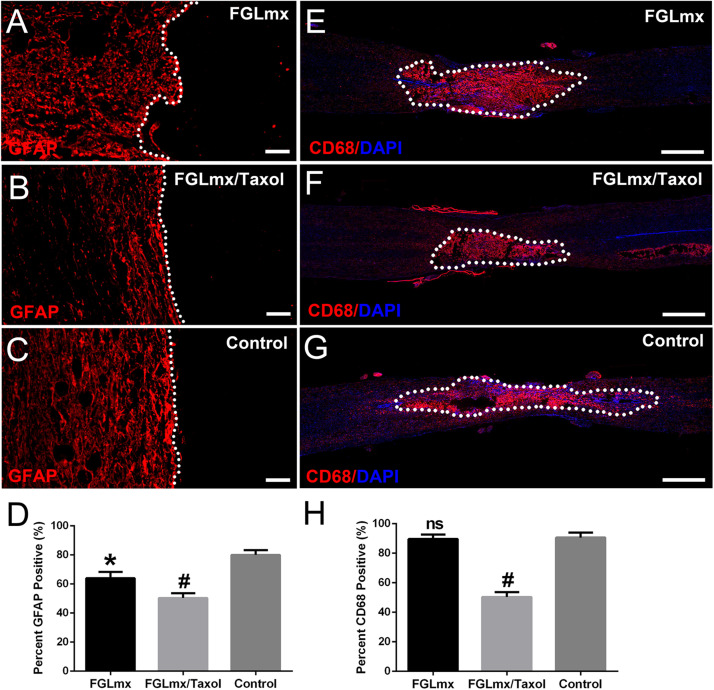
Local delivery of FGLmx/Taxol decreased glial scar formation and inflammation. Images of GFAP staining after treatment with FGLmx **(A)**, FGLmx/Taxol **(B)** and 5% glucose as the control **(C)**. The white dotted line indicates the lesion border. Representative images show longitudinal sections stained with an antibody against CD68 to visualize reactive macrophages (red) and DAPI (blue) in the FGLmx group **(E)**, FGLmx/Taxol group **(F)** and control group treated with 5% glucose **(G)**. Quantification of GFAP **(D)** and CD68 **(H)** staining (± SEM). The scale bar represents 50 μm in images A, B, and C. The scale bar represents 300 μm in images E, F, and G. ^#^*P* < 0.05 compared with the other two groups; *P < 0.05 compared with the control group; ns, not significant.

Astrocyte accumulation was quantified by detecting the density of GFAP staining (Figures 6A–C). A dense GFAP-positive area was observed surrounding the injury site of the control group at 8 weeks after injury. Furthermore, these astrocytes were packed tightly together, resulting in a scar barrier (reactive gliosis). Compared with the control, reactive gliosis was less pronounced and GFAP expression around the injury site was substantially decreased after treatment with FGLmx/Taxol or FGLmx. According to the quantitative analysis, GFAP expression was significantly decreased after the local administration of FGLmx/Taxol (*P* < 0.05), and the reduction induced by FGLmx/Taxol was more substantial than the reduction induced by FGLmx alone (Figure 6D). Thus, the local delivery of FGLmx/Taxol attenuated gliosis at the injury site.

The infiltration of activated inflammatory cells within and around the lesion site is an important signal in chronic inflammation. Staining for CD68, a marker of activated macrophages (Figures 6E–G), was performed with anti-CD68 antibody to assess the activity of macrophages at the lesion site after SCI. FGLmx/Taxol significantly decreased the infiltration of CD68^+^ cells compared to FGLmx or the control at 8 weeks after SCI, confirming the efficacy of this delivery system at inhibiting inflammation after SCI.

### The FGLmx/Taxol Treatment Decreased the Demyelination Area After SCI

**FIGURE 7:**
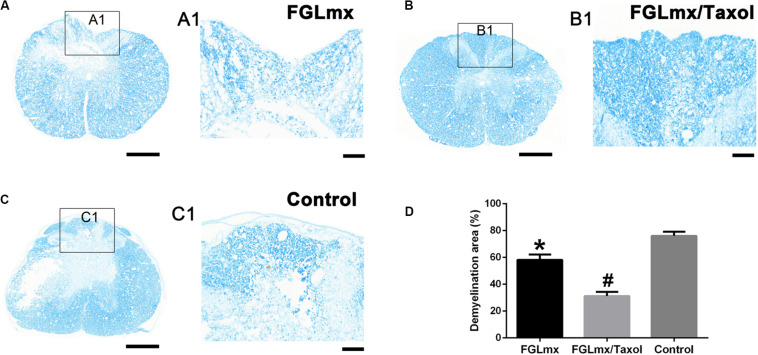
The administration of FGLmx or FGLmx/Taxol reduced demyelination. **(A–C)** Histological assessment of transverse sections using LFB staining at the lesion epicenter. **(A1–C1)** Higher magnification micrographs of the posterior horn. The scale bar represents 100 μm. **(D)** Quantification of the demyelination area in the posterior horn of the lesion epicenter (± SEM). The IOD of the residual myelin sheath was higher in the FGLmx and FGLmx/Taxol groups than in the control group. The scale bar represents 500 μm in (**A–C)** and 100 μm in (**A1,B1,C1)**. ^#^*P* < 0.05 compared with the other two groups; *P < 0.05 compared with the control group.

Luxol fast blue (LFB) staining was conducted to evaluate the effect of FGLmx/Taxol on preserving the myelin sheath. As shown in Figures 7A–C, abundant cell debris and degenerated axons existed near the epicenter of transverse injury in the spinal cord sections. Greater myelin sheath preservation, as assessed by the IOD, was observed in the FGLmx and FGLmx/Taxol groups than in the 5% glucose group (Figure 7D). Compared with the FGLmx group, the FGLmx/Taxol group displayed greater preservation of the myelin sheath. Importantly, we conducted double staining for axons (NF200) and myelin (MBP) to determine the effects of the various treatments on the myelin sheath (Supplementary Figure S1). Based on these results, the local administration of FGLmx/Taxol decreased demyelination in rats after SCI.

### Local Delivery of FGLmx/Taxol Reduced the Cavity Size and Promoted Functional Recovery After SCI

**FIGURE 8:**
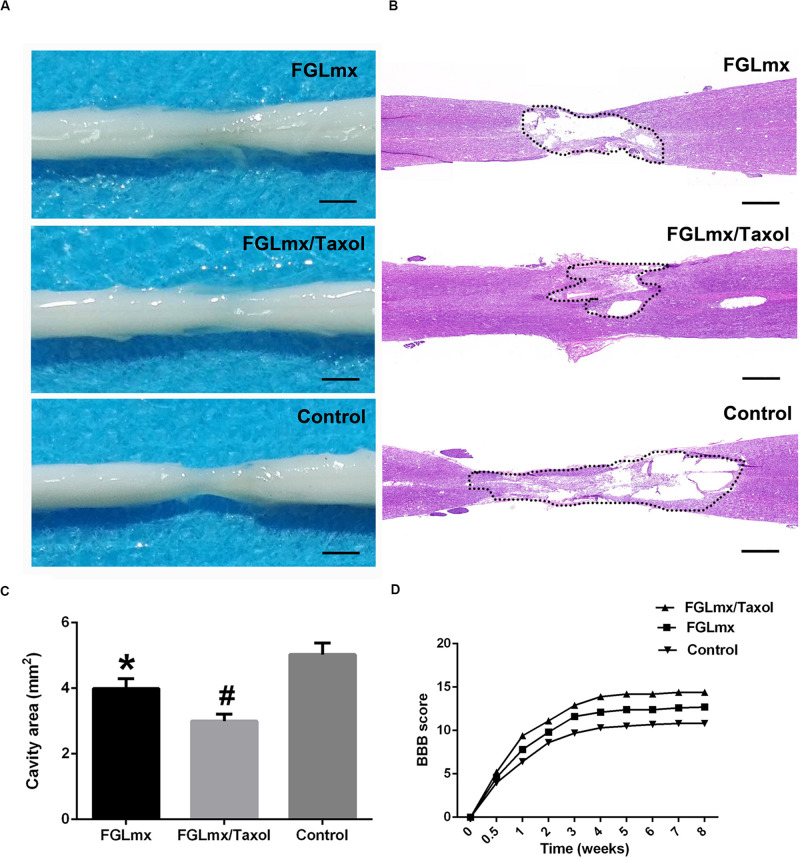
Local administration of FGLmx/Taxol reduced the cavity size *in vivo* and facilitated functional recovery in rats. **(A)** General morphology of the harvested spinal cord after 8 weeks. **(B)** H&E-stained sections of the injury epicenter showing the widespread loss of gray matter and white matter at 8 weeks after contusion injury. **(C)** Quantification of the spinal cord cavity size (± SEM). The cavity area was significantly decreased in the FGLmx/Taxol group compared with the other groups. **(D)** BBB scores. The scale bar represents 500 μm. ^#^*P* < 0.05 compared with the other two groups; *P < 0.05 compared with the control group.

Eight weeks after SCI, we measured the extent of the cavity and resected the injured spinal cord from the experimental animals for a histological assessment of the cavity that formed (Figure 8A). Longitudinal sections were stained with H&E to investigate the effect of FGLmx/Taxol on the cavity dimension (Figure 8B). As shown in Figure 8C, the average cavity area of the longitudinal sections was 5.12 ± 0.35 mm^2^ in the control group compared to 3.10 ± 0.28 mm^2^ in the FGLmx/Taxol group and 3.95 ± 0.24 mm^2^ in the FGLmx group. The FGLmx/Taxol and FGLmx groups showed smaller lesion areas than the control group, but this difference was more pronounced in the FGLmx/Taxol group, suggesting that the injection of FGLmx/Taxol after SCI reduced cavity formation.

The recovery of hindlimb function was evaluated weekly by two blinded examiners using the BBB scale to assess the effects of the scaffold injected into the lesion site on motor function after SCI. All rats were completely paralyzed immediately after the contusion injury and displayed partial recovery during the 1st week. The average BBB scores of the FGLmx and FGLmx/Taxol groups were significantly higher than the control group (Figure 8D). Moreover, higher BBB scores were observed in the FGLmx/Taxol group than in the FGLmx group (Supplementary Video S2). Thus, Taxol exerted a positive effect on rats after SCI, and treatment with FGLmx/Taxol promoted the recovery of hindlimb function in rats after SCI.

## Discussion

In a previous study, we certified that FGLmx was a good candidate for neuroengineering and rehabilitation, as it increased the adhesion and survival of SC-NSCs *in vitro* (Wang et al., 2015). The results of the current study suggest that Taxol released from FGLmx/Taxol retains its bioactivity. Additionally, we confirmed that the release of Taxol from the nanofiber scaffold can be controlled. Finally, the administration of the Taxol delivery system, i.e., FGLmx/Taxol, after SCI is associated with increased neurite extension, reduced cavity formation, decreased demyelination, and reduced inflammation and astrocyte accumulation at the lesion site, which together eventually promote neurobehavioral and functional recovery in rats after SCI. In summary, the local delivery of Taxol from an FGL-functionalized SAP nanofiber scaffold has potential for use in preclinical studies.

The dynamic rearrangements of microtubules are essential for axonal regeneration after axotomy (Das and Miller, 2012; Kleele et al., 2014; Ruschel et al., 2015). At lower concentrations, Taxol promotes polymerization at the plus end and alters microtubule dynamics by limiting disorganized microtubules to a chaotic morphology, eventually inhibiting the formation of retraction bulbs after axotomy (Sengottuvel et al., 2011). However, limited by the narrow treatment window, the administration of Taxol should be controlled to promote the extension of growth cones instead of microtubule overstabilization and avoid neuronal toxicity. The present study addresses these concerns by loading Taxol into a self-assembling nanofiber scaffold to apply Taxol at lower concentrations than the concentrations applied in previous studies (Fan et al., 2018).

Previous methods of Taxol administration for SCI therapy include osmotic minipumps (PerezEspejo et al., 1996) and modified collagen scaffolds (Fan et al., 2018; Yin et al., 2018). In our study, we devised the FGL-functionalized SAP nanofiber scaffold, i.e., FGLmx, for Taxol administration. The innovative vehicle avoids the dilution of Taxol in a neuropathic solvent, directs the growth of a permissive nanofiber scaffold to promote neurite extension across the injury site, is biocompatible and allows long-term drug release. By loading Taxol into the hydrogel nanofiber scaffold, neurons were exposed to Taxol via controlled release, which promoted neurite extension by stabilizing microtubules.

The reactive astrogliosis that usually occurs after SCI includes the release of inhibitory molecules that potentially inhibit axonal outgrowth (Yao et al., 2018). The inhibitory effect of astrogliosis is alleviated by applying growth-promoting treatments (Tysseling-Mattiace et al., 2008). In our study, the administration of FGLmx decreased the accumulation of astrocytes surrounding the injury site, even at 8 weeks after SCI. Because astrocytes are the predominant cells that inhibit axonal outgrowth, mainly by secreting CSPGs and forming a glial scar, strategies that decrease the accumulation of astrocytes at the injury site may alleviate these effects, thus promoting the potential regeneration of neurites through the glial scar border. Furthermore, the combination of these potential effects with the controlled release of Taxol may have been responsible for the observed increase in the nerve fiber density.

In the current study, FGLmx/Taxol-treated animals displayed greater cell infiltration into the injury site, resulting in a decreased inflammatory response, as determined by CD68^+^ immunostaining for macrophages at the lesion site. This finding is consistent with previous reports describing that the substitution of the injured tissue with functionalized SAPs promotes cell infiltration into the injury site (Rodriguez et al., 2014; Hassannejad et al., 2019).

Overall, the local delivery of Taxol from an FGL-functionalized nanofiber scaffold is achieved in a controlled manner over a long period, and the Taxol released from FGLmx/Taxol retains its bioactivity and promotes neurite extension *in vitro*. Moreover, the localized administration of FGLmx/Taxol increases neurite extension across the lesion border, inhibits pathological glial activation and inflammation, decreases demyelination, reduces cavity formation and promotes neurobehavioral recovery. These findings are promising because the local delivery of Taxol via a biodegradable drug delivery system implanted at the lesion site exerts beneficial therapeutic effects on repair after SCI.

## Data Availability Statement

The raw data supporting the conclusions of this article will be made available by the authors, without undue reservation, to any qualified researcher.

## Ethics Statement

The animal study was reviewed and approved by the Animal Experimentation Ethics Committee of Huazhong University of Science and Technology.

## Author Contributions

ZX, YY, and ZW conducted the SCI experiment model, injection of drug and spinal cord dissections, and wrote the manuscript. QT performed the primary cortical neurons cultures *in vitro*. ZX, JW, LG, and BL performed the data collection and analyses. YW and QZ conceptualized the study and took responsibility of acquiring funding for the work. All authors contributed to the article and approved the submitted version.

## Conflict of Interest

The authors declare that the research was conducted in the absence of any commercial or financial relationships that could be construed as a potential conflict of interest.
