# Supplementary material for: Local Delivery of Taxol From FGL-Functionalized Self-Assembling Peptide Nanofiber Scaffold Promotes Recovery After Spinal Cord Injury
Source: Front Cell Dev Biol. 2020 Aug 21;8:820. doi: 10.3389/fcell.2020.00820 (PMC7471253; doi:10.3389/fcell.2020.00820)
Supplement: Supplementary file 1 [file Data_Sheet_1.pdf]

Supplementary Material

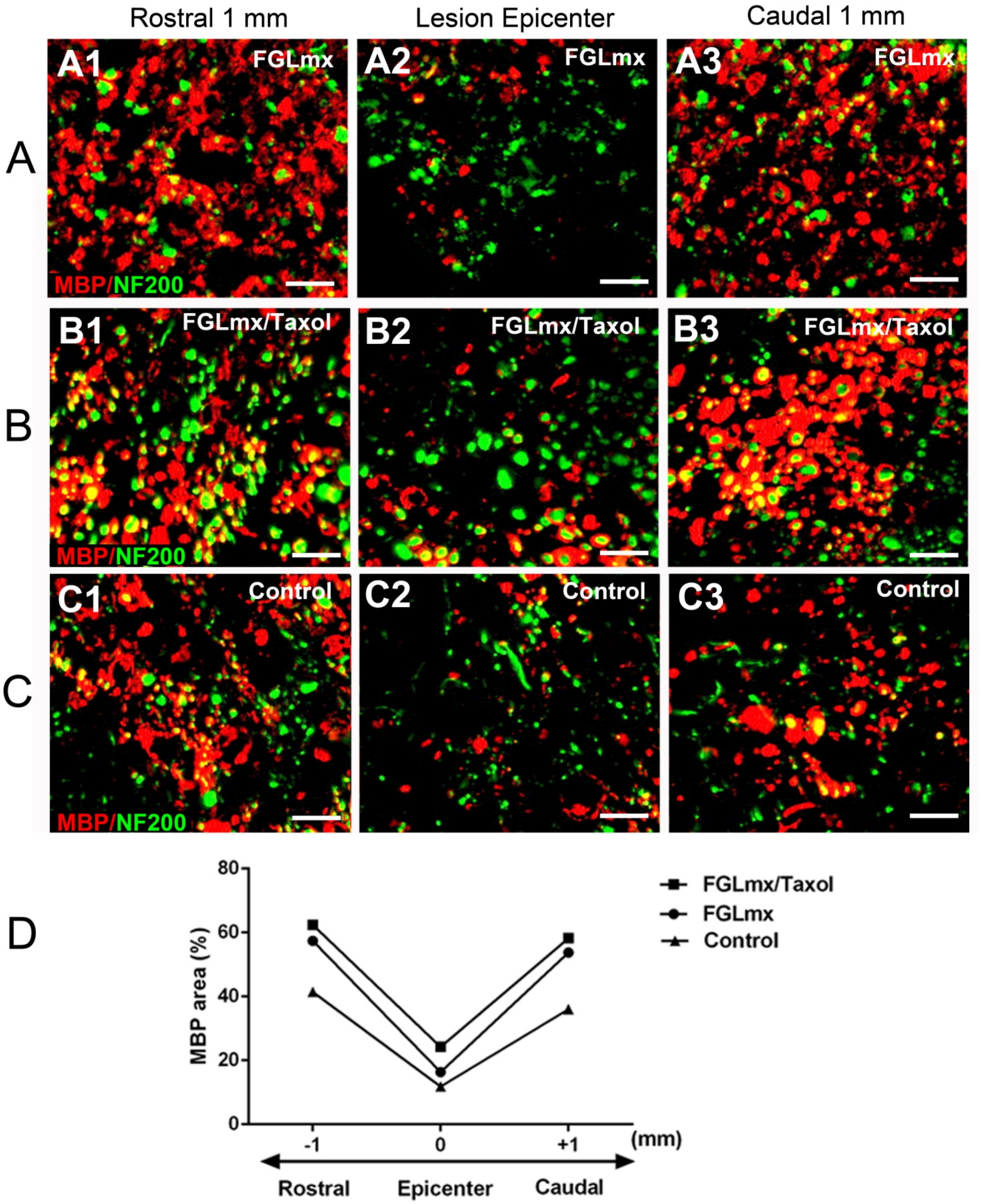

FIGURE S1 | Remyelination of dorsal column axons at the lesion site is frequently associated with oligodendrocytes. Costaining for axons (NF200; green) and myelin (MBP; red) illustrating remyelinating oligodendrocytes in the dorsal column of the spinal cord at the lesion site at 8 weeks after injury. **(A)** FGLmx-treated group. **(B)** FGLmx/Taxol-treated group. **(C)** Control group. The scale bars are 25 µm. **(D)** Quantification of dorsal column axons with remyelination in serial sections of 2-mm spinal cord segments incorporating the injury epicenter. The x-axis refers to the distance from the injury epicenter.

VIDEO S2 | Behavioral Assessments.

Time	Video	Groups	The Average BBB Score	Neurobehavioral Features
The day after injury	Video 1	FGLmx	0	No observable hindlimb (HL) movements were observed
	Video 2	FGLmx/Taxol	0	
	Video 3	Control	0	
2 weeks after injury	Video 4	Control	7	Extensive movement of all three HL joints was observed
	Video 5	FGLmx	9	Plantar placement with weight support in stance only or consistent weight supported dorsal stepping and no plantar stepping was observed
	Video 6	FGLmx/Taxol	11	Frequent to consistent weight-supported steps and no forelimb-hindlimb coordination was observed
8 weeks after injury	Video 7	Control	9	Plantar placement with weight support in stance only or frequent weight supported dorsal stepping and no plantar stepping was observed
	Video 8	FGLmx	11	Frequent to consistent weight-supported steps and no forelimb-hindlimb coordination was observed
	Video 9	FGLmx/Taxol	14	Consistent coordinated plantar stepping, predominant paw position is rotated at initial contact and liftoff; Frequent plantar stepping, consistent forelimb-hindlimb coordination, and occasional dorsal stepping was observed

TABLE S3 | The BBB Scale (Basso et al., 1996).

Score	Characteristics	Comment
0	No observable hindlimb (HL) movements	
1	Slight movement of one or two HL joints	Slight = ≤ 50% of joint range
2	Extensive movement of one HL joint and possible slight movement of one other joint	Extensive = > 50% of joint range
3	Extensive movement of two HL joints	Two joints = usually hip and knee
4	Slight movement of all three HL joints	Three joints = usually hip, knee and ankle
5	Slight movement of two HL joints and extensive movement of HL third joint	
6	Extensive movement of two HL joints, slight movement of third HL joint	Third joint = usually the ankle
7	Extensive movement of all three HL joints	
8	Sweeping with no weight support or Plantar placement with no weight support	Sweeping = rhythmic extensions of three HL joints, rat on side
9	Plantar placement with weight support in stance only or Occasional, frequent or consistent weight supported dorsal stepping and no plantar stepping	Weight support = contraction of HL extensor muscle during plantar placement of paw or elevation of hindquarters in stance only
10	Occasional weight-supported steps with no forelimb-hindlimb (FL-HL) coordination	Occasional = >5% and ≤ 50%;<br>Steps = plantar contact with weight support, HL advances to reestablish plantar contact;<br>Coordination = one HL step per FL step, alternating HL steps
11	Frequent to consistent weight-supported steps and no FL-HL coordination	Frequent = 51- 94% of the time;<br>Consistent = 95 - 100% of time
12	Frequent to consistent weight-supported steps and occasional FL-HL coordination	6-50% bouts of coordinated locomotion
13	Frequent to consistent weight-supported steps and frequent FL-HL coordination	51-95% bouts of coordinated locomotion
14	Consistent coordinated plantar stepping, predominant paw position is rotated at initial contact and liftoff; Frequent plantar stepping, consistent FL-HL coordination, and occasional dorsal stepping	Rotated = internal or external rotation of the hind paw when placed or at liftoff
15	Consistent coordinated plantar stepping, no or occasional toe clearance during forward limb advancement, predominant paw position is parallel at initial contact	Parallel = hind paw placement parallel to body on initial contact or liftoff;<br>Toe clearance = listen for toe drag sounds, i.e., footsteps without toe drag sound
16	Consistent coordinated plantar stepping, frequent toe clearance, and predominant paw position is parallel at initial contact and rotated at lift-off	Frequent toe clearance = more than half of footsteps have no toe drag sounds
17	Consistent coordinated plantar stepping, frequent toe clearance, predominant paw position is parallel at initial contact and lift-off	
18	Consistent coordinated plantar stepping, consistent toe clearance, predominant paw position is parallel at	Consistent toe clearance = ≤ 4 toe drag sounds during the 4-min observation period

	initial contact and lift-off	
19	Consistent coordinated plantar stepping, consistent toe clearance, predominant paw position parallel at initial contact and lift-off, tail is down part or all the time	Tail down = tail touches ground during stepping
20	Consistent coordinated plantar stepping, consistent toe clearance, predominant paw position parallel at initial contact and lift-off, tail consistently up, and trunk instability	Tail up = does not touch ground;<br>Trunk instability = lateral weight shifts when turning quickly, waddling, lurching, falling
21	Consistent coordinated gait, consistent toe clearance, predominant paw position is parallel at initial contact and lift-off, tail consistently up, and consistent trunk stability	Consistent trunk stability: no lurching or falling; pelvic girdle and tail in line with locomotion

Basso, D. M., Beattie, M. S., Bresnahan, J. C., Anderson, D. K., Faden, A. I., Gruner, J. A., et al. (1996). MASCIS evaluation of open field locomotor scores: effects of experience and teamwork on reliability. Multicenter Animal Spinal Cord Injury Study. *J Neurotrauma*, *13*(7), 343-359. doi: 10.1089/neu.1996.13.343
